# Isoimperatorin Reduces Synovial Inflammation and Fibrosis in Knee Osteoarthritis via the cAMP Signalling Pathway

**DOI:** 10.1111/jcmm.70880

**Published:** 2025-10-06

**Authors:** Lishi Jie, Junnan Liu, Zaishi Zhu, Zeling Huang, Yujiang Liu, Guanhong Liu, Xiaofeng Shen, Yuwei Li, Xiaoqing Shi

**Affiliations:** ^1^ Key Laboratory for Metabolic Diseases in Chinese Medicine, First College of Clinical Medicine, Nanjing University of Chinese Medicine Jiangsu Provincial Hospital of Traditional Chinese Medicine Nanjing China; ^2^ Department of Orthopaedics and Traumatology, Affiliated Hospital of Nanjing University of Chinese Medicine Jiangsu Provincial Hospital of Traditional Chinese Medicine Nanjing China; ^3^ Department of Orthopaedics and Traumatology Suzhou TCM Hospital Affifiliated to Nanjing University of Chinese Medicine Suzhou China

**Keywords:** cAMP signalling pathway, fibrosis, isoimperatorin, RNA‐seq, synovial inflammation

## Abstract

To explore the mechanism of pharmacological action of Isoimperatorin (ISO), a small molecule compound with anti‐inflammatory properties extracted from the rhizome of *Notopterygium incisum*, in attenuating synovial inflammation in knee osteoarthritis (KOA). By establishing a rat model of KOA and using histopathology and molecular biology methods, we evaluated the pharmacological effect of ISO on synovitis. Synovial fluid from the knee joint was collected for transcriptomic and metabolomic analyses. Fibroblast‐like synoviocytes were cultured in vitro, and calcium fluorescence imaging and mitochondrial membrane potential assays were performed to assess the effects of ISO on the cAMP signalling pathway and KOA‐related synovial inflammation. Preliminary pharmacodynamic observations showed that ISO was able to reduce synovial inflammation in KOA rats. Further transcriptomic findings in synovial tissues indicated that the mechanism of action of ISO was related to the cAMP signalling pathway and calcium ion signalling pathway. The results of metabolomics showed that the progression of synovial fibrosis was related to the abnormal metabolism of glycerophospholipids, and the intervention of ISO could significantly promote the metabolism of glycerophospholipids in synovial tissues. Finally, the results of in vitro experiments showed that ISO improved the level of inflammation and the degree of fibrosis in synovial cells, activated the cAMP signalling pathway and promoted PPAR expression, whereas inhibition of the activation of the cAMP signalling pathway attenuated the effects of ISO. ISO promotes PPAR function by upregulating the cAMP signalling pathway to modulate glycerophospholipid metabolism, thereby alleviating synovial inflammation and slowing fibrosis progression in KOA.

**Abbreviations**
cAMP: cyclic adenosine monophosphate
DMEM: Dulbecco's modified eagle medium
FLS: fibroblast‐like synoviocytes
H&E: haematoxylin & eosin
IL‐15: interleukin‐15
IL‐1β: interleukin‐1β
IL‐6: interleukin‐6
ISO: isoimperatorin
KOA: knee osteoarthritis
NF‐κB: nuclear factor‐κB
PMSF: phenylmethylsulfonyl fluoride
TNF‐α: tumour necrosis factor

## 1 Introduction

Knee osteoarthritis (KOA) is a common chronic joint disease, the incidence of which increases significantly with age, especially in the elderly population, where the prevalence of KOA can be as high as 40%–60% in people over 65 years of age [1]. In addition, factors such as gender, obesity, occupation and heredity all play a role in the risk of developing the disease, with women usually more susceptible to KOA than men due to changes in hormone levels after menopause [2, 3]. In recent years, the incidence of KOA has been on the rise due to changes in lifestyle and the ageing of the population, posing a huge challenge to public health and the social care system [4]. Therefore, in‐depth research on KOA will help develop more effective treatments to alleviate social pressure and improve the quality of life of patients.

Synovial inflammation is one of the important pathological features of KOA, which further promotes the degeneration of intra‐articular cartilage and alteration of bone structure by increasing the proliferation of synovial fibroblasts with the release of inflammatory mediators [5, 6]. Studies have shown that synovial inflammation not only affects patients' joint function and quality of life, but is also closely related to the severity of KOA pain [6]. At the same time, it has been shown that persistent synovial inflammation is a key factor in the development of synovial fibrosis, and that the release of inflammatory mediators not only contributes to the proliferation and activation of fibroblasts, but also affects the expression of matrix metalloproteinases, which exacerbates the fibrotic process [7]. Exploring the pathogenesis of synovial inflammation and searching for safe and effective measures to control the progression of synovial inflammation in a timely manner will help us to treat KOA more effectively.

Integrated analysis of metabolomics and transcriptomics has become a key strategy in systems biology. By simultaneously mapping the landscape of gene expression and metabolites, this multi‐omics approach comprehensively reveals the full regulatory network from gene transcription to metabolic phenotype. In recent years, this combined technique has been widely applied in medicine, agriculture and microbiology. In osteoarthritis research, integrative analysis can identify critical metabolic pathways and their upstream transcriptional regulators, offering new avenues for drug target discovery. With the incorporation of artificial intelligence and machine learning, multi‐omics data integration is advancing towards greater intelligence and precision, providing robust technical support for unravelling complex biological phenomena.

Isoimperatorin (ISO), which is widely found in *Angelica dahurica*, *Notopterygium incisum* and other Chinese herbal medicines, has attracted a great deal of attention in inflammatory diseases in recent years as a small molecule bioactive ingredient [8, 9]. Relevant studies have shown that ISO possesses significant anti‐inflammatory effects and can reduce the production of pro‐inflammatory factors by inhibiting the activation of the nuclear factor‐κB (NF‐κB) signalling pathway, thus effectively reducing inflammatory responses [10]. In addition, ISO has been found to improve chondrocyte function, promote cartilage matrix synthesis and inhibit apoptosis, which are important for the prevention and treatment of KOA [11]. However, the role ISO plays in the treatment of KOA is not yet clear, and we ISO has been found to act as an acetylcholinesterase may play a more critical role in ameliorating the pain process. A large number of peripheral nerves are widely present in the synovial tissue of the knee to sense pain [12, 13, 14]. Thus, we focused on ISO treatment of KOA in synovial tissue to observe the pharmacological effects of ISO.

By using transcriptomics and metabolomics in this study, we attempted to further explore and investigate the potential drug mechanism of action of ISO to ameliorate the synovial inflammatory and fibrotic processes of KOA, which will provide more diverse horizons and directions for the subsequent treatment of KOA.

## 2 Methods and Materials

### 2.1 Animals

Male Sprague–Dawley rats weighing 200–220 g were obtained from the Animal Center of the Nanjing University of Traditional Chinese Medicine. The rats were bred and housed in the Experimental Animal Center of Nanjing University of Traditional Chinese Medicine. The raising conditions included a room temperature of 22°C–26°C and a relative humidity of 40%–70%. They were treated according to the National Institutes of Health Guidelines for the Care and Use of Laboratory Animals. All experiments were approved and supervised by the Laboratory Animal Welfare Ethics Committee of Suzhou Institute of Biomedical Engineering and Technology, Chinese Academy of Sciences (Approval No. 2024‐A92).

### 2.2 KOA Rat Model

Fifty rats were randomly divided into the following three groups: sham group (*n* = 10), KOA group (*n* = 10) and ISO treatment group (*n* = 30). A modified Hulth method was used to build the KOA model, as in our previous study. Briefly, after anaesthesia (isoflurane 1.5%–2%), animals underwent surgery. The right knee joint cavity of 40 rats was exposed by a parapatellar medial incision. We cut the medial collateral ligament and anterior cruciate ligament of model rats, and then resected the medial meniscus. Finally, we sutured the incision in layers. In the control/sham group, rats were only exposed to the knee joint cavity. For KOA treatment, ISO (HY‐N0286, 99.61% purity, MCE Corporation, USA) was administered orally, and the dose was calculated based on previous studies using the dosage conversion formula between humans and rats. The equivalent dose coefficient conversion formula indicated that the low dose for rats was 25 mg/kg, the medium dose was 50 mg/kg and the high dose was 100 mg/kg. After that, we sutured these incisions. Animals in the control/sham and KOA groups also received the same dose of 0.9% saline.

### 2.3 Enzyme Linked Immunosorbent Assay

After 4 weeks of treatment, the animals were administered anaesthesia (isoflurane, 1.5%–2%), blood was collected from the cava and the serum was prepared in a centrifuge (10,000 rpm, 30 min, 4°C). Interleukin‐1β (IL‐1β), Interleukin‐6 (IL‐6), Interleukin‐15 (IL‐15) and tumour necrosis factor (TNF‐α) were examined with an ELISA kit (BioTek, USA and Cloud‐Clone Corp, China) according to the manufacturer's instructions.

### 2.4 Histopathology

The rat synovial tissue was fixed with 4% paraformaldehyde fixative. The samples were dehydrated with a gradient ethanol series and embedded in paraffin. Synovial tissue sections with a thickness of 5 μm were carefully prepared, stained with haematoxylin & eosin (H&E), Masson staining and Picrosirius red (Sorabio, China) in sequence according to the kit instructions. The Krenn synovitis score assesses three parameters—synovial lining layer hyperplasia, density of inflammatory cell infiltration and activation of resident cells—each rated from 0 to 3, for a total score of 0 to 9. Based on the total score, synovitis is classified into two grades: low‐grade inflammation (0–4) and high‐grade inflammation (5–9).

### 2.5 Western Blot Analyses

Standard western blot analysis was used to detect the protein expression of COL I (1:1000, 14695‐1‐AP, Proteintech, China), FN1 (1:1000, 15613‐1‐AP, Proteintech, China), TGF‐β (1:1000, 21898‐1‐AP, Proteintech, China) and PAI‐1 (1:1000, 13801‐1‐AP, Proteintech, China) in synovial tissue; cAMP (1:1000, bs‐12707R, Bioss, China), PKA (1:1000, bs‐0520R, Bioss, China), phosphor‐CREB (1:1000, bs‐5270R, Bioss, China), PPAR‐δ (1:1000, 60193‐1‐Ig, Proteintech, China) and PPAR‐γ (1:1000, 16643‐1‐AP, Proteintech, China) in FLS (Fibroblast‐like synoviocytes) and tissue. GAPDH (1:5000, 60004‐1‐Ig, Proteintech, China) was used as the reference protein. Briefly, tibial plateau synovial tissue was ground with liquid nitrogen, weighed and then mixed with RIPA lysis buffer and phenylmethylsulfonyl fluoride (PMSF) (R: *p* = 100: 1) mixed in a 10:1 ratio. The samples were shaken overnight at 4°C and centrifuged at 14,000 rpm for 30 min. The supernatant was collected. Protein quantification was performed using the Dichondroitin Acid Protein Assay Kit (BCA Kit, China). In our experiments, protein grayscale was quantified using ImageJ software.

### 2.6 qRT‐PCR

Synovial tissue from the knee joint was thoroughly homogenised with an electric homogeniser, and total RNA was extracted using the TRIzol method: add 200 μL chloroform and mix, centrifuge and collect the supernatant, precipitate with isopropanol, wash with 75% ethanol and dissolve in DEPC‐treated water. After measuring RNA concentration and purity with a NanoDrop, use 1 μg of total RNA to synthesise cDNA by reverse transcription with the PrimeScript RT kit. Quantitative real‐time PCR was performed using the SYBR Green method. Primer sequences are shown in Table 1.

**TABLE 1:** Primer sequences used in qRT‐PCR.

Target gene	Forward primer	Reverse primer
IL‐6	TCCAGTTGCCTTCTTGGGAC	GTGTAATTAAGCCTCCGACTTGT
IL‐1β	GAAATGCCACCTTTTGACAGTG	TGGATGCTCTCATCAGGACAG
COL I	GACGCCATCAAGGTCTACTGC	ACGGGAATCCATCGGTCA
TGF‐β1	CTCCCGTGGCTTCTAGTGC	GCCTTAGTTTGGACAGGATCTG
GAPDH	GGAGAAACCTGCCAAGTATGATG	GCTGGGACATTGAAAGTCTC

### 2.7 Transcriptomics

Freshly extracted sham, koa and high‐concentration ISO synovial tissues were lysed using Trizol reagent. After successful extraction, RNA was dissolved by adding 50 μL of DEPC‐treated water. Subsequently, total RNA was identified and quantified using a Qubit fluorescence quantifier and a Qsep400 high‐throughput biofragment analyser. A total of three sets of synovial tissue samples were sequenced. The gene expression level of each sample was analysed using HTSeq (v0.5.4p3) software, and the model was set to union. GO enrichment analysis was performed using GOseq (v1.22). KEGG analysis identified key biochemical metabolic pathways and signalling pathways of differentially expressed genes through pathway significance enrichment. For samples with biological replicates, differential expression analysis was performed using DESeq (v1.10.1). The P values of differential expression analysis were corrected for false discovery rate (FDR) using the Benjamini and Hochberg method. The standard for differential gene screening is generally: *p* < 0.05.

### 2.8 Metabolomics

Metabolites were extracted from serum samples of each group, and rat serum was processed and analyzed using a high‐resolution LC–MS/MS system. Raw data were processed using Progenesis QI software, and all mass deviations were kept below 100 ppm. Subsequent studies included normalization of initial peak area data, repeatability assessment within each sample group and exploration of identified compounds in the KEGG and HMDB databases. The screening criteria for differential metabolites were set as *p* < 0.05 and VIP > 1. These parameters were coordinated with those used in similar studies. KEGG enrichment analysis and topoisomerism analysis were also performed.

### 2.9 Correlation Analysis Between Transcriptomics and Metabolomics

The quantitative values of genes and metabolites in all samples were used for correlation analysis. The correlation method was to use the cor function in R to calculate the Pearson correlation coefficient of genes and metabolites, and select the correlation coefficient with an absolute value greater than 0.8 and *p* < 0.05. In each difference group, the correlations with an absolute value of the Pearson correlation coefficient greater than 0.8 and *p* < 0.05 were screened. Subsequently, the difference in the genes and metabolites corresponding to these correlations was displayed through a nine‐quadrant diagram.

### 2.10 Extraction, Culture and Grouping of Rat Synovial Fibroblasts

Primary FLS were isolated from male Sprague–Dawley rats weighing approximately 140–160 g. After euthanasia by cervical dislocation, synovial tissue was collected, rinsed with PBS, minced and digested in DMEM containing type I collagenase (2 mg/mL) for 3 h. The cell suspension was filtered, centrifuged, pelleted and resuspended, then cultured in complete medium (DMEM +10% foetal bovine serum +1% antibiotics). Cells were maintained and passaged in a humidified incubator at 37°C with 5% CO_2_. FLS at passages 3–5 were used for subsequent experiments. To model the condition seen in KOA, FLS were treated with DMEM/F12 containing LPS at 1 μg/mL for 24 h. ISO was then administered at low, medium and high doses of 10, 25 and 50 μM, respectively, for combined intervention.

### 2.11 Cell Counting Kit‐8 (CCK‐8) Assay

Cell viability was determined by CCK‐8 colorimetry. Briefly, FLS were seeded into 96‐well plates with 1 × 10^4^ cells/well and gradient concentrations of ISO (0, 5, 10, 25, 50, 75 and 100 μM) were added. The cells were cultured in a 5% CO_2_ incubator at 37°C for 48 h. Cell viability in each well was quantified by measuring the absorbance at 450 nm using a microplate reader.

### 2.12 Calcium Imaging

According to the protocol provided by the reagent supplier (S1060, Beyotime, China), the Fluo4‐AM stock solution was diluted 1000‐fold with PBS, added to the well plates containing cells, incubated in the dark at 37°C for 30 min, centrifuged at 1800 rpm for 5 min and discarded the supernatant. The cells were washed with PBS, and fluorescence intensity was detected on a microscope, and the relative fluorescence intensity of the calcium ions was calculated. The cells were washed with PBS, and the fluorescence intensity was measured on a microscope.

### 2.13 JC‐1

The functional condition of mitochondria was detected using the JC‐1 staining Kit (C2003S, Beyotime, China) under the instructions of the manufacturer. Briefly, after washing with cold PBS, the FLS cells were added to 1 mL of JC‐1 staining solution and incubated in an incubator (37°C, 5% CO_2_) for 30 min. Then, 2 mL of complete medium was added to the FLS cells. The fluorescence of JC‐1 was detected using a fluorescence microscope (Olympus, Japan).

### 2.14 The Level of ATP (Adenosine Triphosphate)

FLS cells were seeded in 6‐well plates at 1 × 10^5^ cells and treated with IOS, and then operated according to the instructions of the ATP detection kit (bc0300, Sorabio, China).

### 2.15 Statistical Analysis

The experiments in this study included at least three replicates. All results were analyzed using GraphPad Prism 8.0 (San Diego, USA) and expressed as mean ± standard deviation (SD). Two‐way and one‐way analysis of variance (ANOVA) methods were used to compare the conditions of samples in each group at different time points.

## 3 Results

### 3.1 ISO Improves Synovial Inflammation Level and Fibrosis in KOA Rats

**FIGURE 1:**
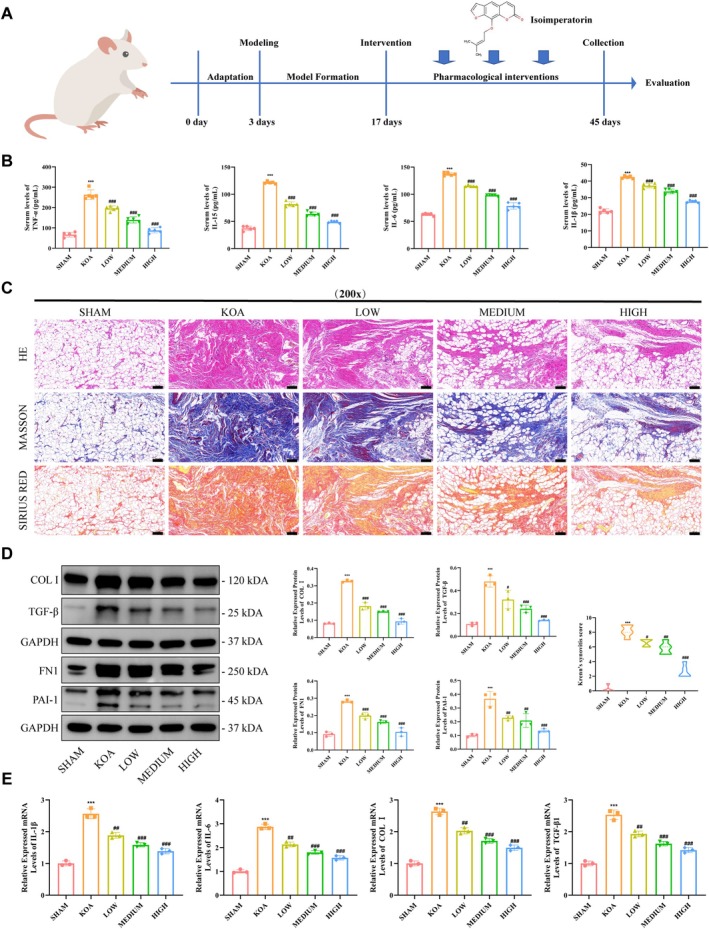
ISO improves synovial inflammation level and fibrosis in KOA rats. (A) Schematic diagram of the animal experimental procedure; (B) ELISA to detect the level of inflammatory factors within the serum of mice in each group (*n* = 5); (C) representative images of histopathological staining of synovial membrane (*n* = 6); (D) WB to detect the level of expression of fibrosis‐associated proteins in synovial membrane tissues; (E) PCR detection of inflammation‐ and fibrosis‐related gene expression levels in synovial tissue. Statistical results were represented by mean ± standard deviation (Mean ± SD, *n* = 3), ***p < 0.001 versus Sham group; ^#^ *p* < 0.05, ^##^ *p* < 0.01, ^###^ *p* < 0.001 versus KOA group.

By administering ISO at three dose gradients to KOA rats, we observed that ISO alleviated synovial inflammation associated with KOA. The in vivo experimental workflow is shown in Figure 1A. Serum levels of inflammatory cytokines IL‐1β, IL‐6, IL‐15 and TNF‐α were measured by ELISA, and the results showed that ISO reduced inflammatory cytokine levels in KOA rats, with efficacy positively correlated with dose. Histopathological staining of synovial tissues in each group indicated that ISO progressively improved synovial fibrosis as the concentration increased, with a corresponding decrease in Krenn scores. Further, Western blot analysis of fibrosis‐related proteins in synovial tissues demonstrated that, compared with the control group, protein expression levels of FN1, COL I, TGF‐β and PAI‐1 were elevated in the KOA group; in contrast, KOA rats receiving ISO exhibited decreased expression of FN1, COL I, TGF‐β and PAI‐1, showing a dose‐dependent effect across the concentration gradient. PCR results showed that, relative to the control group, mRNA expression of IL‐1β, IL‐6, COL I and TGF‐β in KOA synovial tissue was significantly upregulated, whereas ISO intervention reduced the mRNA expression of IL‐1β, IL‐6, COL I and TGF‐β in synovial tissue in a concentration‐dependent manner.

### 3.2 Metabolomics Analysis of the Effect of ISO on Serum Metabolites in KOA Rats

**FIGURE 2:**
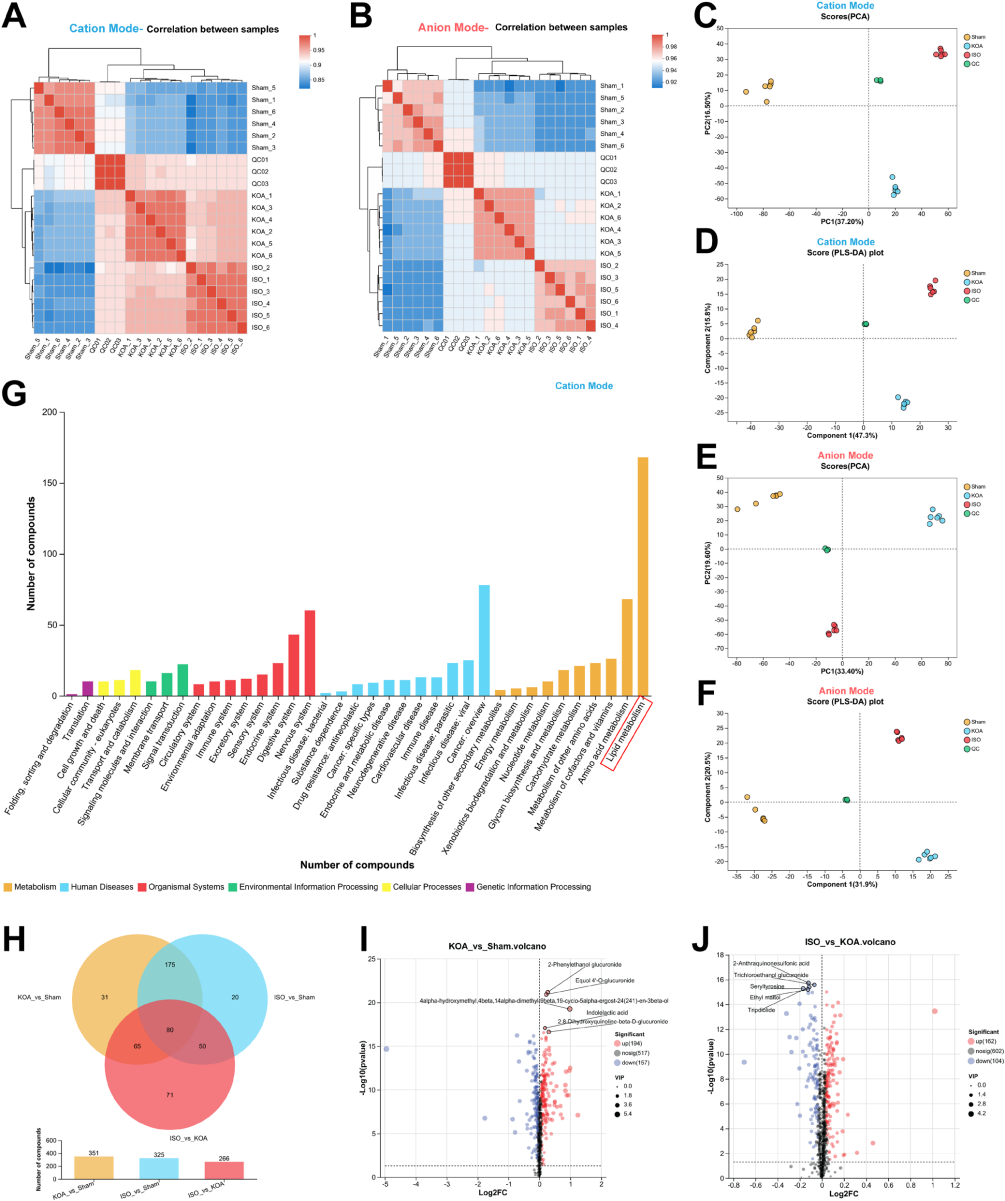
Metabolomics analysis of the effect of ISO on serum metabolites in KOA rats. (A) Heatmap of correlation difference between samples of each group in cation mode; (B) Heatmap of correlation difference between samples of each group in anion mode; (C) PCA analysis of samples of each group in cation mode; (D) PLS‐DA analysis of samples of each group in cation mode; (E) PCA analysis of samples of each group in anion mode; (F) PLS‐DA analysis of samples of each group in anion mode; (G) Identification of differential metabolite categories based on the HMDB database to identify differential metabolite classes; (H) KEGG database‐based identification of differential metabolite classifications; (I) Venn diagrams demonstrating the number of differential metabolites under two‐by‐two comparisons of samples from the three groups; (J) volcano scatter plots demonstrating the number of differential metabolites between the KOA group and the SHAM group; and (I) volcano scatter plots demonstrating the number of differential metabolites between the ISO group and the KOA group.

To further validate the mechanism of action of ISO in ameliorating fibrosis and inflammation in KOA synovial tissue, we used metabolomics to analyse the changes of relevant metabolites in the serum of the sham‐operated group, the KOA group and the ISO high‐concentration group. The results of the correlation analysis between individual samples in cationic and anionic modes showed (Figure 2A,B) that we found significant differences in metabolites between the three groups. The significant differences between the metabolites among the three groups were further supported by PCA analysis and PLS‐DA analysis (Figure 2C–F). By comparing the results of the HMDB database (Figure 2G), the metabolites differing among the three groups mainly focused on phospholipid metabolites. The comparison of the KEGG database also showed that the metabolites were mainly concentrated in phospholipid metabolism (Figure 2H). The results of the differential metabolites between the three groups in a two‐by‐two comparison shown by the Venn diagram (Figure 2I) showed that there were 351 differential metabolites in the sham‐operated group compared with the KOA group, of which 31 were unique differential metabolites; while there were 266 differential metabolites in the ISO group compared with the KOA group, of which 71 were unique differential metabolites. Further, the changes in differential metabolites between the two groups were demonstrated by volcano plots, which showed (Figure 2J,K) that there were 194 metabolites with increased expression and 157 differential metabolites with decreased expression during KOA compared to the sham operation group, whereas there were 162 metabolites with increased expression and 104 differential metabolites with decreased expression after the ISO intervention compared to the KOA group.

### 3.3 ISO Treatment Promotes Glycerophospholipid Metabolic Pathways in KOA Rats

**FIGURE 3:**
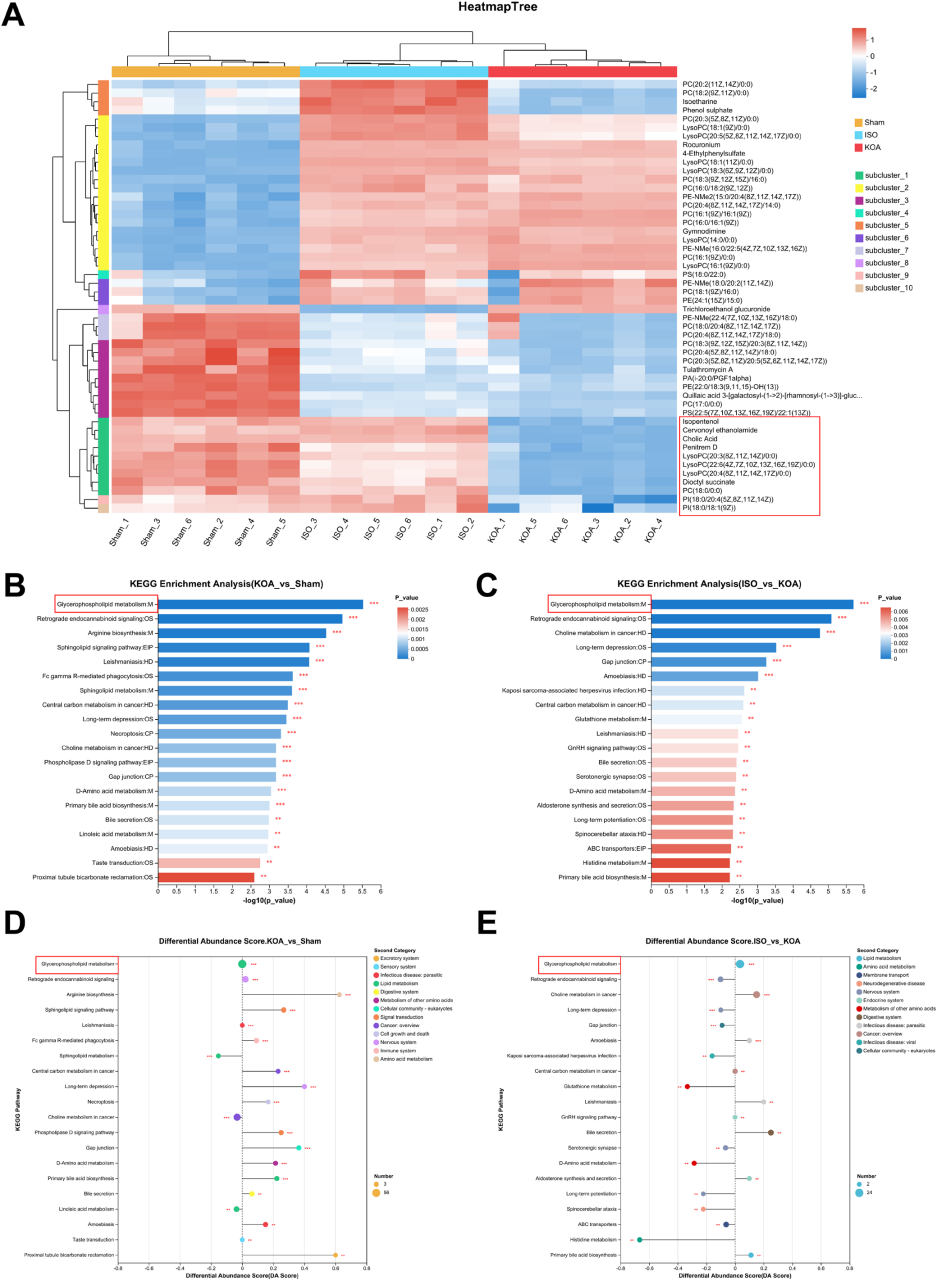
ISO treatment promotes glycerophospholipid metabolic pathways in KOA rats. (A) Subgroup clustering heat map to analyse differential metabolites within each group of samples; (B) KEGG enrichment analysis of differential metabolic pathways between the KOA and SHAM groups; and (C) KEGG enrichment analysis of differential metabolic pathways between the ISO and KOA group for differential metabolic pathways; (D) abundance difference scores for differential metabolic pathways between the KOA and SHAM groups; (E) abundance difference scores for differential metabolic pathways between the ISO and KOA groups.

Subgroup clustering heatmap analysis of serum metabolites against the three groups showed (Figure 3A) that the KOA procedure resulted in increased expression levels of lsoetharine and phenol sulphate, while the ISO intervention decreased the metabolic levels of lsoetharine and phenol sulphate. Further results of PCA analysis, PLS‐DA analysis, permutation testing and OPLS‐DA for serum samples from the KOA group versus sham operation group showed significant differences in metabolites between the two groups (Figure S1A); while PCA analysis, PLS‐DA analysis for serum samples from the ISO group versus KOA group, permutation testing and OPLS‐DA showed significant differences in metabolites between the two groups (Figure S1B). The results of differential metabolites between the KOA group and the sham‐operated group analysed by VIP showed (Figure S1C) that the levels of metabolites such as carnosol and humulone were reduced in the KOA group as compared to the sham‐operated group, whereas the levels of haloxyfop, 6‐ethyichenodeoxycholic acid and 27‐hydroxyisomangiferolic acid metabolite levels were increased. Analysis of the differential metabolite results between the KOA group and the sham operation group by VIP showed (Figure S1D) that the metabolite levels of phaseic acid and hydroxyphenyfacetyiglycine were decreased in the ISO group compared to the KOA group, whereas the metabolite levels of 2,5,7‐trihydroxy‐4′‐methoxyisoflavanone and 7‐ketodeoxycholic acid metabolite levels were increased. The main differential metabolic pathways in the KOA group and the sham‐operated group were determined by KEGG enrichment analysis and abundance difference scores (Figure 3B,D), in which the results of the enrichment analysis mainly focused on glycerophospholipid metabolism. The main differential metabolic pathways in the KOA group and the sham‐operated group were determined by KEGG enrichment analysis and abundance difference scores (Figure 3C,E), and the results of KEGG enrichment analysis after ISO intervention also focused on glycerophospholipid metabolism. Meanwhile, we performed KEGG topology analysis, which also revealed that the glycerophospholipid metabolism process plays a key role in the generation of KOA disease (Figure S1E), according to the results demonstrated in the KEGG pathway, in which metabolites such as cardiolipin, phosphatidylglycerol and other metabolites occurred changed (Figure S1D). After ISO intervention, glycerophospholipid metabolism was the metabolic process with the most significant changes (Figure S1F), and it was able to significantly reduce the levels of metabolites such as phosphatidylglycerol, phosphatidylethanolamine, etc. (Figure S1H).

In summary, it is reasonable to judge that the glycerophospholipid metabolic pathway in the phospholipid metabolism process was mainly targeted after ISO intervention.

### 3.4 Transcriptomic Analysis of the Effect of ISO on Synovial Tissue in KOA Rats

**FIGURE 4:**
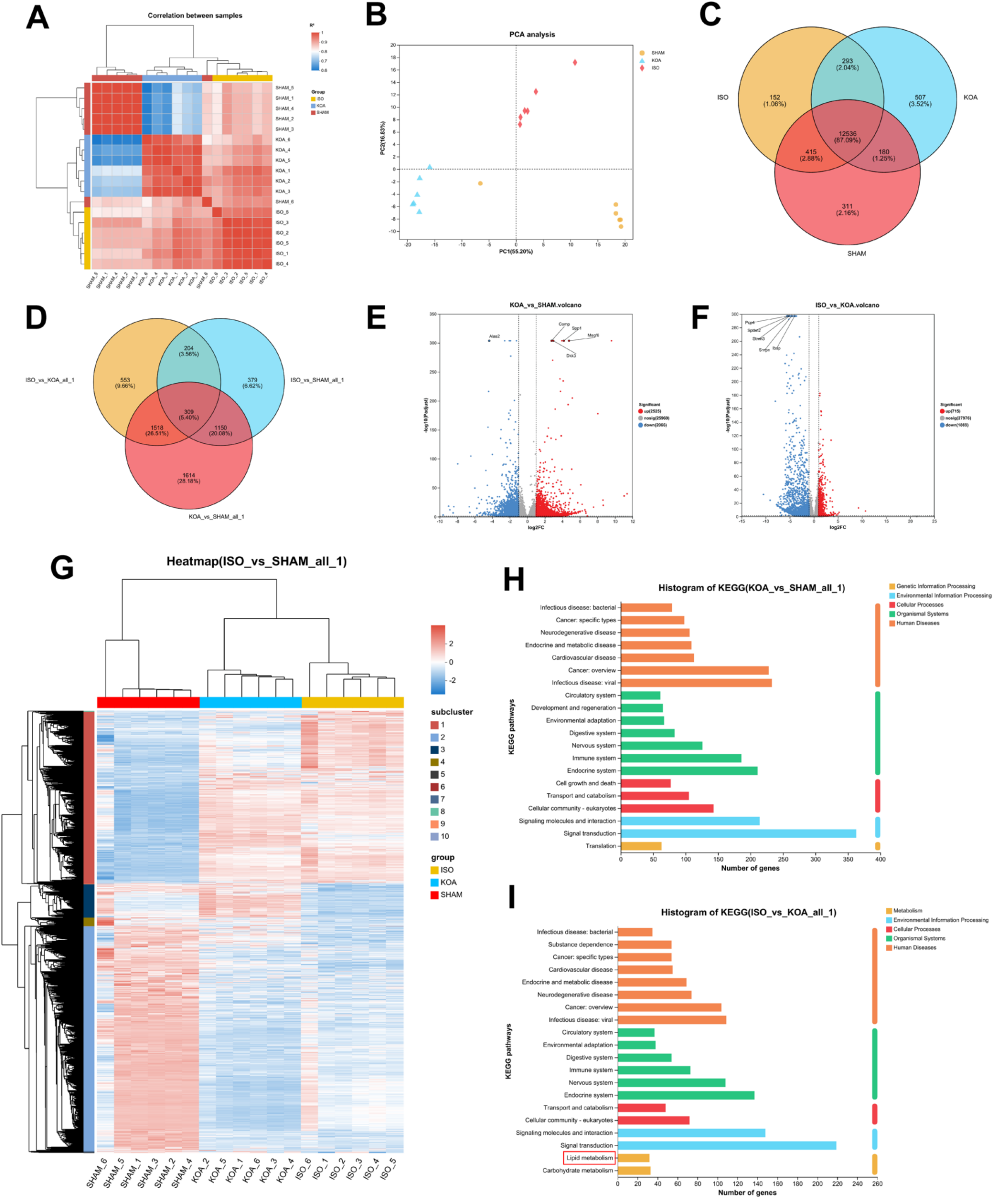
Transcriptomic analysis of the effect of ISO on synovial tissue in KOA rats. (A) Correlation heatmap between synovial tissue samples from each group; (B) PCA analysis of synovial tissue samples from each group; (C) Venn diagram showing the number of differential genes between the three groups of samples; (D) Venn diagram showing the number of differential genes under two‐by‐two comparison of samples from the three groups; (E) Volcano scatter plot showing the number of differential genes between the KOA group and the SHAM group; (F) Volcano scatter plot showing the number of differential genes between the ISO group and the KOA group; (G) Subgroup clustering heatmap analysing differential genes within each group of samples; (H) KEGG annotation analysing the differential gene pathways between the KOA group and the SHAM group; (I) KEGG annotation analysing the differential gene pathways between the ISO group and the KOA group.

By using RNA‐Seq to analyze the differences in transcript levels in the sham‐operated group, KOA group and the ISO high concentration group, the results of the inter‐sample correlation heatmap showed (Figure 4A) that the differences in transcript levels between the sham‐operated group and the KOA were significant, and that the synovial tissues after the ISO intervention more closely resembled the sham‐operated group. The results of the PCA analysis (Figure 4B) also confirmed that the ISO‐intervened Venn diagram (Figure 4C) demonstrated a total of 12,536 co‐expressed genes among the three groups, of which the sham‐operated group contained 311 uniquely expressed genes, the KOA group contained 507 uniquely expressed genes, and the ISO group contained 152 uniquely expressed genes. The Venn diagram was further used to show the number of differential genes after two‐by‐two comparisons among the three groups (Figure 4D), in which 4591 differential genes existed between the sham‐operated group and the KOA group, of which 1614 differential genes were specific differential genes between the two groups; whereas 2584 differential genes existed between the ISO group and the KOA group, of which 553 differential genes were specific differential genes between the two groups. By volcano plot analysis (Figure 4E,F), there were 2525 differential genes with increased expression levels and 2066 differential genes with decreased expression levels between the KOA group compared to the sham operation group; and there were 715 differential genes with increased expression levels and 1869 differential genes with decreased expression levels between the ISO group compared to the KOA group. The results of differential gene clustering analysis showed (Figure 4G) that there were significant differences in differential genes between the KOA group and the sham‐operated group, whereas there was partial similarity between the differential genes and the sham‐operated group after the ISO intervention. Further analysis of the difference in differential genes between the KOA group and the sham operation group and between the ISO group and the KOA group using the KEGG database to classify the differential genes (Figure 4H,I) showed that there was a significant enrichment of Environmental Information in the differential genes between the KOA group and the sham operation group and between the ISO group and the KOA group. Processing classification of Signalling molecules and interaction and Signal transduction. At the same time, the metabolic changes after the ISO intervention were mainly focused on lipid metabolism, which coincided with the results of metabolomics.

### 3.5 ISO Treatment Modulates cAMP Signalling Pathway in Synovial Tissue of KOA Rats

**FIGURE 5:**
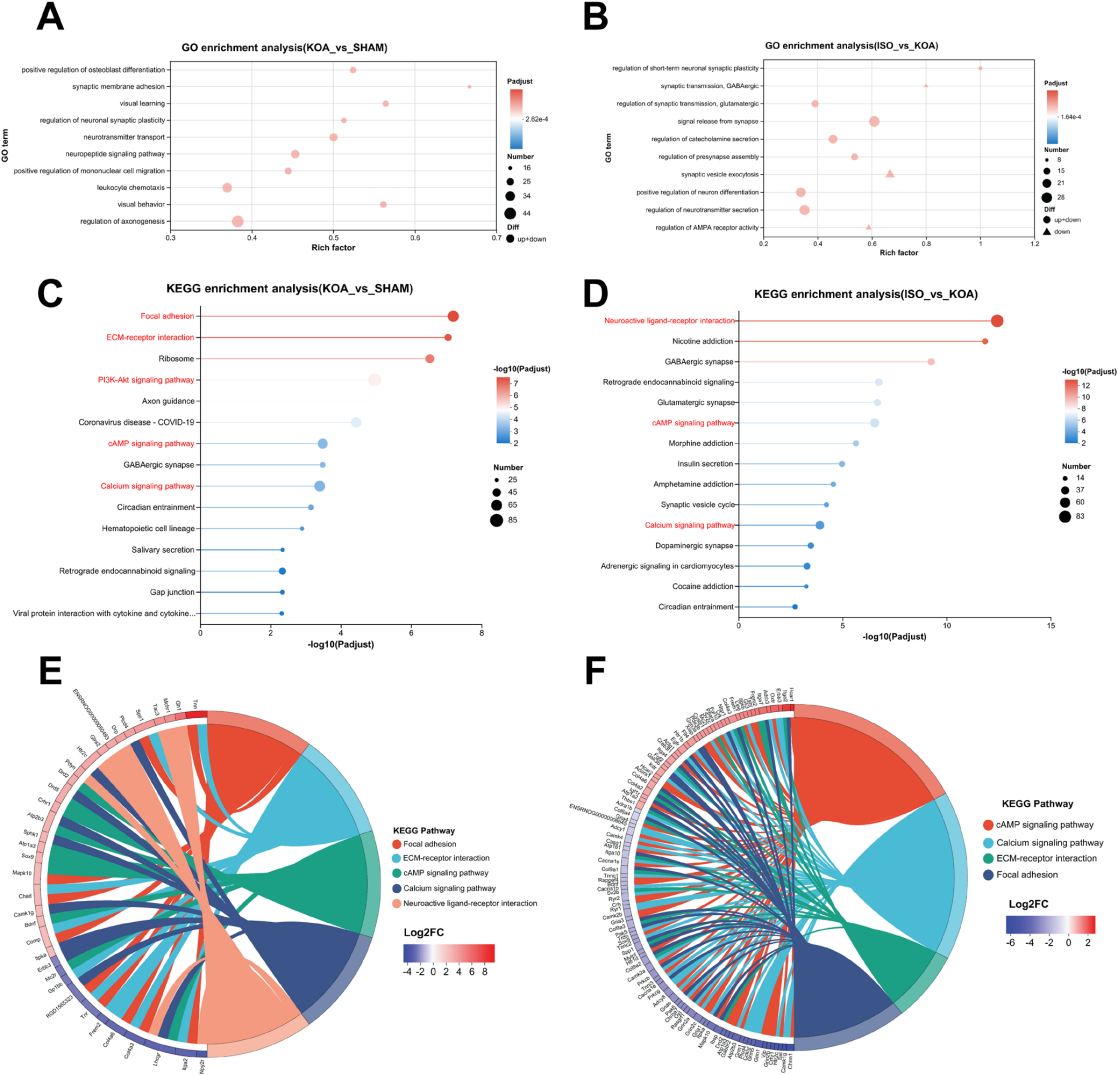
ISO treatment modulates cAMP signalling pathway in synovial tissue of KOA rats. (A) GO enrichment analysis of differential signalling pathways between synovial tissue samples of KOA and SHAM groups; (B) GO enrichment analysis of differential signalling pathways between synovial tissue samples of ISO and KOA groups; (C) KEGG enrichment analysis of the differential signalling pathway between synovial tissue samples of KOA and SHAM groups; (D) KEGG enrichment analysis of the differential signalling pathway between synovial tissue samples of ISO and KOA groups; (E) KEGG enrichment chordal graphs demonstrating the differential signalling pathway between synovial tissue samples of KOA and SHAM groups; (F) KEGG‐enriched chordal graphs showing the major differential genes within the differential signalling pathways between the synovial tissue samples from the ISO and KOA groups.

The results of differential gene analysis using GO enrichment for the two subgroups showed (Figure 5A,B) that the differential genes between the KOA group and the sham‐operated group mainly focused on positive regulation of osteoblast differentiation, synaptic membrane adhesion, neurotransmitter transport and positive regulation of mononuclear cell migration, which are related to the progression of synovial tissue inflammation and fibrosis; whereas after ISO intervention, the differential genes mainly focused on the regulation of short‐term neuronal synaptic plasticity, synaptic transmission, GABAergic and other metabolic remodelling related terms. Meanwhile, KEGG enrichment analysis showed (Figure 5C,D) that the cAMP signalling pathway was co‐enriched by differential genes between the KOA group and the sham‐operated group and between the ISO group and the KOA group. We further focused on some of the internal changes in signalling pathways that appeared to be significantly altered during the KOA process and ISO intervention, which were demonstrated using KEGG enrichment analysis chordal plots (Figure 5E,F). The results showed that the levels of genes related to focal adhesion and ECM‐receptor interaction, such as TNN and SPP1, were significantly up‐regulated during KOA, while ISO intervention significantly reduced the expression levels of these genes. Meanwhile, we found that the expression levels of cAMP signalling pathway and calcium signalling pathway genes were significantly altered during KOA, whereas the intervention of ISO negatively regulated the changes in cAMP signalling pathway and calcium signalling pathway genes caused by the KOA process.

### 3.6 Joint Analysis of Transcriptomics and Non‐Target Metabolomics

**FIGURE 6:**
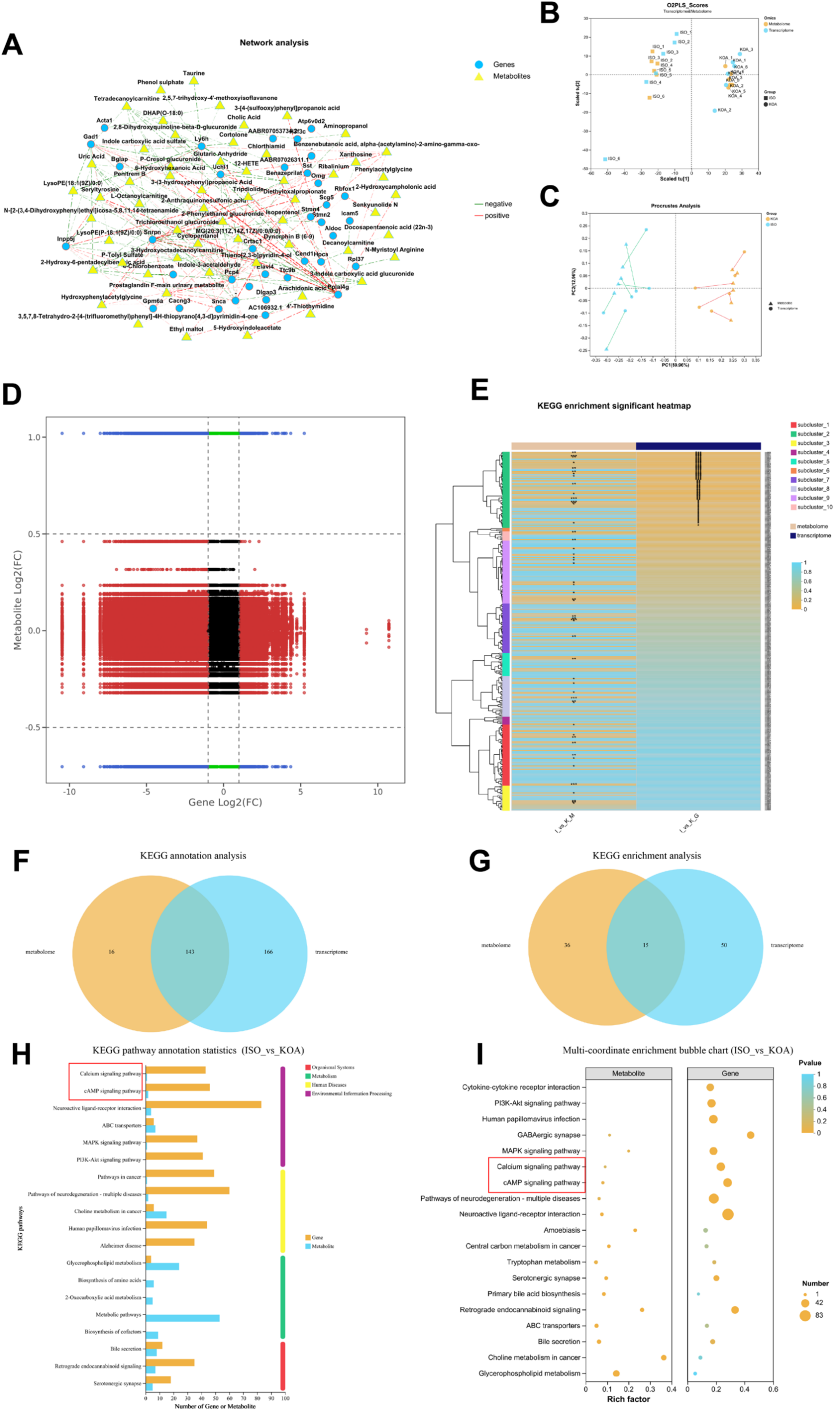
Joint analysis of transcriptomics and non‐target metabolomics. (A) Correlation network plot demonstrating potential linkage between differential genes and differential metabolites; (B) O2PLS scoring of concordance between differential genes and differential metabolites; (C) Procrustes analysis demonstrating linkage between differential genes and differential metabolites; (D) Nine‐quadrant plot demonstrating variable relationship between differential genes and differential metabolites; (E) KEGG subpopulation clustering enrichment analyses association between differential genes and differential metabolites; (F) Venn diagram demonstrating KEGG annotation analysis of differential genes and differential metabolites intersection; (G) Venn diagram demonstrating KEGG enrichment analysis of differential genes and differential metabolites intersection; (H) KEGG annotation analysis of differential genes and differential metabolites signalling pathways; (I) KEGG enrichment analysis of differential genes and differential metabolites signalling pathways.

Combining the results of RNA‐seq and metabolomics to further clarify the potential mechanism of action of ISO in ameliorating synovial inflammation and fibrosis, firstly, a correlation network diagram was drawn based on the potential links between differential genes and differential metabolites (Figure 6A). The O2PLS score and Procrustes analysis were used to determine whether there was concordance between the differential genes and metabolites between the ISO and KOA groups (Figure 6B,C), and the results showed that there was similarity between the metabolites and genes within both the ISO and KOA groups. Changes between differential genes and differential metabolites were further refined using a nine‐quadrant plot (Figure 6D), which showed that differential genes and metabolites were much higher in quadrants 1, 2 and 4 than in quadrants 6, 8 and 9, and thus it is reasonable to assume that the ISO intervention regulates the transcriptional process of differential genes more. Analysis of differential genes and differential metabolites by KEGG enrichment showed (Figure 6E), while there was a significant correlation between differential genes and differential metabolites after ISO intervention. The KEGG annotation analysis showed (Figure 6F) that there were 143 pathways contained in the differential metabolites and 143 pathways contained in the differential genes, and the statistical graph of KEGG annotation analysis (Figure 6H) revealed that the differential metabolites and differential genes were concentrated in the cAMP signalling pathway and calcium ion signalling pathway. And by KEGG enrichment analysis showed (Figure 6G) that there were 15 pathways enriched in differential metabolites and 15 pathways enriched in differential genes. And as shown by multi‐coordinate KEGG enrichment analysis (Figure 6I), the cAMP signalling pathway and calcium ion signalling pathway may be the key signalling pathways for ISO to exert drug action. In conclusion, it is reasonable to believe that ISO exerts its pharmacological effects to improve synovial inflammation and fibrosis through the cAMP signalling pathway and calcium ion signalling pathway.

### 3.7 ISO Treatment Activates Calcium Inward Flow to Promote cAMP/PKA/CREB Signalling Pathway

**FIGURE 7:**
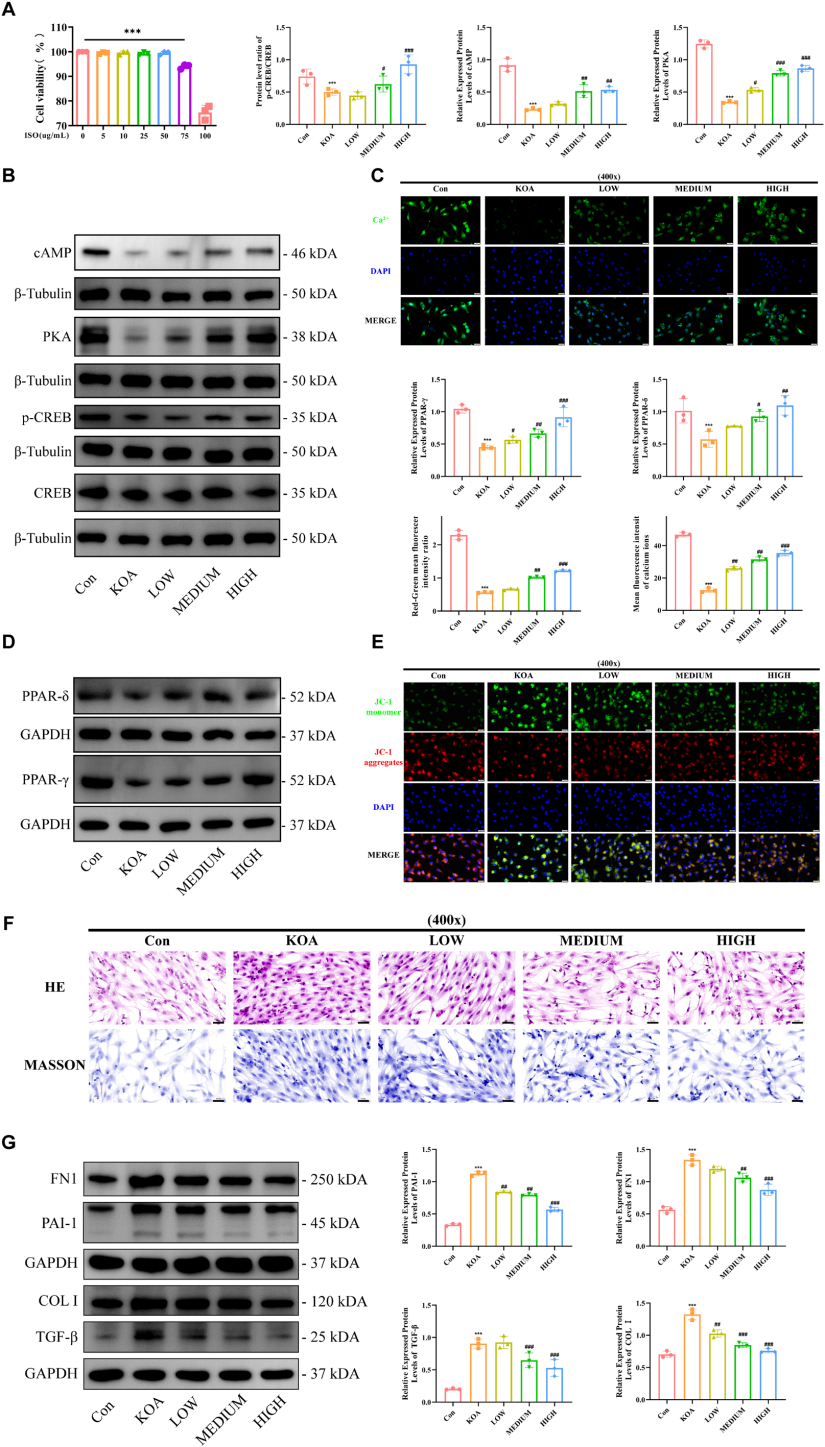
ISO treatment activates calcium inward flow to promote cAMP/PKA/CREB signalling pathway. (A) CCK‐8 to detect cell viability; (B) WB to detect the expression level of relevant proteins in the cAMP pathway in cells; (C) Calcium ion detection kit to observe the intensity of calcium ion signals in cells; (D) WB to detect the protein expression level of PPAR‐γ and PPAR‐δ in cells; (E) JC‐1 kit to observe the level of mitochondrial membrane potential in cells; (F) pathological staining to observe the cell morphological changes; (G) WB detection of fibrosis‐related protein expression level in cells; Statistical results were represented by mean ± standard deviation (Mean ± SD, *n* = 3), ***p < 0.001 versus control group; ^#^ *p* < 0.05, ^##^ *p* < 0.01, ^###^ *p* < 0.001 versus KOA group.

In order to verify the results of transcriptome and metabolome, we observed the pharmacological effects of ISO by intervening rat primary synovial fibroblasts using ISO. Firstly, we screened the suitable concentration of ISO by CCK‐8 assay, and the results showed that (Figure 7A) the survival rate of synovial fibroblasts began to decrease when the concentration of ISO was greater than 75 μg/mL. And then the WB experiment was used to verify the effect of ISO on the protein expression levels of cAMP and PKA and the phosphorylation level of CREB in synovial fibroblasts, and the results showed that (Figure 7B), compared with the blank group, the protein expression levels of cAMP and PKA and the phosphorylation level of CREB in the synovial fibroblasts of KOA simulated by LPS were decreased; and the synovial fibroblasts were treated with the intervention of ISO. cAMP and PKA protein expression levels and CREB phosphorylation levels were up‐regulated in the synovial fibroblasts treated with ISO intervention, and the drug of ISO showed a concentration gradient. Simultaneously using the observation of calcium fluorescence level of the cells in each group showed (Figure 7C), compared with the blank group, the intensity of calcium fluorescence in synovial fibroblasts decreased in KOA synovial fibroblasts; whereas, the intensity of calcium fluorescence in synovial fibroblasts was up‐regulated after the ISO intervention treatment. Further, we observed the expression level of PPAR, a key protein downstream of CREB phosphorylation, and the mitochondrial membrane potential level (Figure 7D,E), which showed that the protein expression level of PPAR‐δ and PPAR‐γ and the mitochondrial membrane potential level decreased in KOA synovial fibroblasts compared to the blank group, while the protein expression level of PPAR‐δ and PPAR‐γ decreased in synovial fibroblasts after ISO intervention treatment. PPAR‐γ protein expression levels and mitochondrial membrane potential levels were up‐regulated, and the drug of ISO showed a concentration gradient. At the same time, we also observed the effect of ISO on the fibrotic process in synovial fibroblasts, the results of pathological staining showed that (Figure 7F), KOA synovial fibroblasts showed obvious fibrotic changes, with an increase in cell density and a decrease in the cell gap, while ISO intervention showed a more obvious anti‐fibrotic effect, with a reduction in cell density and an increase in the cell gap. The results of WB showed that (Figure 7G), the protein expression levels of TGFBR1, TGFBR2, TGF‐β and PAI‐1 were up‐regulated in the synovial fibroblasts of KOA compared to the blank group; whereas, the protein expression levels of FN1, COL I, TGF‐β and PAI‐1 were down‐regulated after the treatment of ISO intervention, and the drug of ISO showed a concentration gradient. The trend of this result is consistent with the results obtained in in vivo experiments.

### 3.8 cAMP Inhibition Reduces ISO Amelioration of Synovial Fibrosis

**FIGURE 8:**
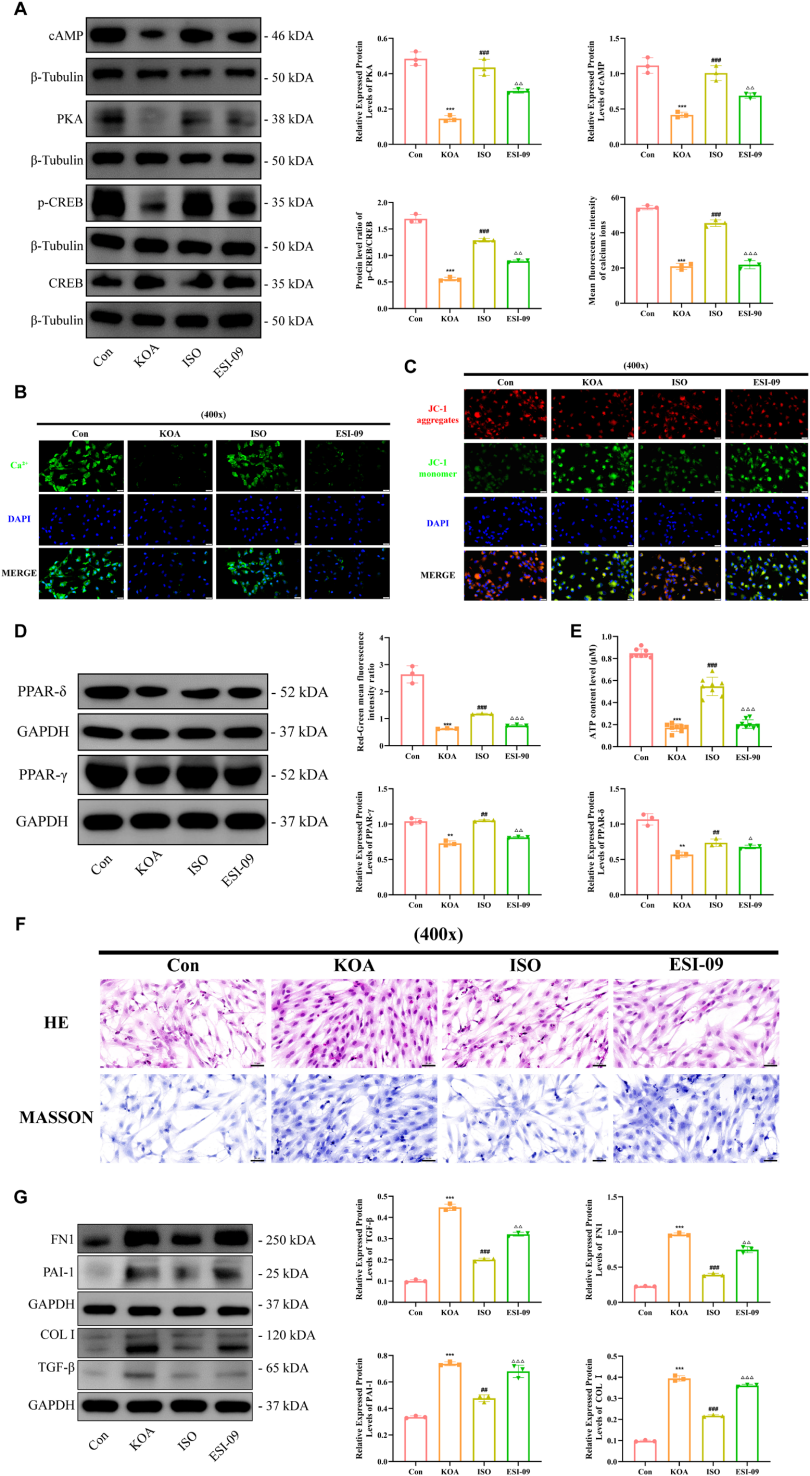
cAMP inhibition reduces ISO amelioration of synovial fibrosis. (A) WB detected the expression level of relevant proteins in cAMP pathway in cells; (B) Calcium ion detection kit observed the intensity of calcium ion signals in cells; (C) JC‐1 kit observed the level of mitochondrial membrane potential in cells; (D) WB detected the expression level of proteins of PPAR‐γ and PPAR‐δ in cells; (E) ATP detection kit observed the level of ATP content in cells; (F) Pathological staining to observe the morphological changes of cells; (G) WB to detect the expression level of fibrosis‐related proteins in cells; Statistical results were represented by mean ± standard deviation (Mean ± SD, *n* = 3), **p < 0.01, ***p < 0.001 versus control group; ^##^ *p* < 0.01, ^###^ *p* < 0.001 versus KOA group; ^△^ *p* < 0.05, ^△△^ *p* < 0.01, ^△△△^ *p* < 0.001 versus ISO group.

We further verified the mechanism of drug action of ISO by using ESI‐09, an inhibitor of cAMP. ESI‐09 inhibits the cAMP signalling pathway by competing with PKA. The results of WB showed (Figure 8A) that ISO intervention treatment up‐regulated the levels of cAMP and PKA protein expression and the phosphorylation level of CREB in synovial fibroblasts of KOA; whereas, the ESI‐09 intervention attenuated the drug action of ISO and decreased the levels of cAMP and PKA protein expression and the phosphorylation level of CREB in synovial fibroblasts. cAMP and PKA protein expression levels and CREB phosphorylation levels in synovial fibroblasts. The results of calcium ion imaging showed (Figure 8B) that ISO intervention treatment up‐regulated calcium ion fluorescence intensity in KOA synovial fibroblasts, while ESI‐09 intervention attenuated the drug effect of ISO. We observed that ESI‐09 intervention decreased the mitochondrial membrane potential of synovial fibroblasts, eliminated the repairing effect of ISO on mitochondrial membrane potential, and aggravated mitochondrial damage (Figure 8C). ESI‐09 intervention down‐regulated the protein expression levels of PPAR‐δ and PPAR‐γ, and attenuated the promoting effect of ISO on this protein (Figure 8D). And while ESI‐09 intervention reduced the ATP content of synovial fibroblasts, eliminating the repairing effect of ISO on energy metabolism (Figure 8E). In terms of improving fibrosis, the results of pathological staining showed (Figure 8F) that ESI‐09 intervention led to an increase in synovial fibroblast cell density and a decrease in cell gap, which weakened the antifibrotic function of ISO. And the results for fibrosis‐related protein levels showed (Figure 8G) that ESI‐09 intervention up‐regulated the protein expression levels of FN1, COL I, TGF‐β and PAI‐1, which attenuated the anti‐fibrotic drug effect of ISO. Taken together, it is reasonable to suggest that ISO may exert its antifibrotic effects by activating the cAMP signalling pathway.

## 4 Discussion

The synovial inflammatory and fibrotic process plays a key role in the development of KOA and is a major player in the KOA process [5]. The transforming growth factor‐β (TGF‐β) family plays a crucial role in the fibrotic process and is widely present in various types of tissue repair and pathological changes. TGF‐β factors affect the activation, proliferation and secretory functions of fibroblasts through their multiple signalling pathways, thereby promoting the accumulation and remodelling of the extracellular matrix [15, 16, 17]. The researchers found that TGF‐β induced the transformation of fibroblasts to myofibroblasts, which led to their enhanced production of collagen and other matrix components to accelerate the fibrosis process [17]. Meanwhile, PAI‐1 plays a key role in the proliferation of synovial cells, and its elevated concentration tends to be positively correlated with the degree of fibrosis in synovial tissues, and the overexpression of PAI‐1 is thought to be an important factor leading to the abnormal accumulation of extracellular matrix components [18, 19]. Our results showed that ISO was effective in inhibiting the expression levels of PAI‐1, TGF‐β and their receptor proteins in both in vivo and in vitro experiments.

Normal functioning of energy metabolism is essential to maintain the physiological function of cells. Synoviocytes, as a major component of joints, depend on an adequate supply of energy to maintain their normal function [20]. However, it was found that the energy metabolism of synoviocytes was significantly altered in patients with KOA, which was mainly manifested by impaired mitochondrial function and enhanced glycolytic pathways [20, 21]. Disorders of energy metabolism not only directly affect the physiological function of synoviocytes, but also induce inflammation by increasing the production of ROS, leading to intracellular acid–base imbalance [22]. Meanwhile, disturbances in energy metabolism are closely related to changes in glycerophospholipid metabolism. Glycerophospholipids not only act as energy storage molecules in cellular energy metabolism, but also participate in the regulation of metabolic signals [23]. When energy metabolism is disturbed, intracellular glycerophospholipid levels are also affected, which in turn affects the structure and function of the cell membrane [24]. Studies have shown that a decrease in glycerophospholipid levels may lead to an increase in cell membrane permeability, prompting the release of inflammatory factors, thus exacerbating synovial inflammation in KOA.

The intracellular cyclic adenosine monophosphate (cAMP, cyclic adenosine monophosphate) signalling pathway is a widely studied signal transduction pathway that plays an important role in energy metabolism [25]. cAMP is converted from ATP catalysed by adenylate cyclase. When the G protein‐coupled receptor (GPCR) on the cell surface is activated by an exogenous agonist, the α‐subunit of the G protein binds to adenylate cyclase, which in turn contributes to the production of cAMP [26]. The production of cAMP activates protein kinase A (PKA), which regulates cellular functions by phosphorylating target proteins and triggering a series of physiological responses within the cell [27]. It has been found that in liver cells, cAMP activates PKA to enhance hepatic glucose output, thereby increasing blood glucose levels, whereas in muscle cells, cAMP promotes glycogenolysis and provides energy to meet the energy requirements of cellular activities [28]. The cAMP signalling pathway is also closely related to glycerophospholipid metabolism. cAMP is involved in the synthesis and degradation of glycerophospholipids through the regulation of enzymes and transport proteins related to lipid metabolism [29, 30]. In addition, the importance of cAMP in glycerophospholipid metabolism is also reflected in the regulation of the fluidity of biological membranes, which directly affects the activity of proteins such as calmodulin and the efficiency of signal transduction [31].

As a natural compound with multiple biological activities, ISO has strong antioxidant, anti‐inflammatory and anti‐tumour biological activities and exerts its pharmacological effects through various pathways, including the regulation of intracellular signalling and inhibition of free radical generation [32]. In the cardiovascular system, ISO is able to lower blood pressure, improve the contractility of the heart and prevent the occurrence of heart failure [33]. In the field of neurological diseases, ISO is able to combat the occurrence of neurodegenerative diseases by promoting the survival and growth of neuronal cells, showing potential in the treatment of neurological diseases such as Alzheimer's disease and Parkinson's disease [34]. Meanwhile, in recent years, ISO has been found to be able to regulate the level of cAMP in research, thus causing changes in the physiological functions of cells [35, 36].

At the same time, related studies reported that ISO is able to effectively inhibit the proliferation of human lung fibroblasts under specific stimuli and reduce their cell migration ability, thus slowing down the process of fibrosis [37]. ISO application significantly inhibited the expression of the TGF‐β family in fibroblasts and attenuated the TGF‐β signalling to inhibit the progression of fibrosis. In addition to its direct effects on fibroblasts, ISO has also been found to modulate the inflammatory microenvironment, thereby indirectly affecting the development of fibrosis [38]. ISO significantly reduces the expression levels of a variety of inflammatory factors, including IL‐6 and TNF‐α [37]. The rise in these factors is usually accompanied by an increase in the fibrotic process; thus, the anti‐inflammatory effect of ISO provides further support for its anti‐fibrotic effect.

Although this study explores the role of ISO in addressing synovial inflammation and fibrosis, further research is needed on its effects on energy metabolism and glycerophospholipid metabolism. Future work should delve deeper into the mechanisms of ISO to achieve a comprehensive understanding of its pharmacological effects. In addition, this study primarily evaluates the short‐term effects of ISO; it lacks investigations into the efficacy and potential adverse reactions associated with long‐term use, as well as strategies for translating experimental findings into clinical practice. Subsequent research should place greater emphasis on the long‐term safety and effectiveness of ISO to provide more comprehensive evidence for clinical application and actively explore concrete clinical use strategies and dose‐optimization schemes.

In conclusion, in this study, we found that ISO inhibits the expression of TGF‐β and its receptor and exerts anti‐inflammatory and antifibrotic effects through in vivo experiments, and further analyzed the cAMP signalling pathway through the combined analysis of the transcriptome and metabolome, which plays a key role in the intervention of ISO, and we found that ISO activates the calcium channel to promote the expression of cAMP/PKA, which promotes the phosphorylation of CREB for the repair of the synovial membrane, and the phosphorylation of CREB for the repair of the synovial membrane. CREB phosphorylation repaired the mitochondrial function of synovial cells and up‐regulated the expression level of PPAR family proteins to promote energy metabolism and lipid metabolism, which in turn reduced the synovial inflammation of KOA and alleviated synovial fibrosis.

## Author Contributions

**Lishi Jie:** writing – original draft (equal). **Zaishi Zhu:** methodology (equal), resources (equal). **Junnan Liu:** data curation (equal), investigation (equal). **Zeling Huang:** data curation (equal), resources (equal). **Yujiang Liu:** investigation (equal). **Guanhong Liu:** data curation (equal), formal analysis (equal). **Xiaofeng Shen:** project administration (equal). **Yuwei Li:** funding acquisition (equal), writing – review and editing (equal). **Xiaoqing Shi:** funding acquisition (equal), writing – review and editing (equal).

## Consent

The authors have nothing to report.

## Conflicts of Interest

The authors declare no conflicts of interest.

## Supporting information


**FIGURE S1:** (A) PCA analysis, PLS‐DA analysis, Permutation testing and OPLS‐DA to analyse the variability and reliability between samples from the KOA and SHAM groups; (B) PCA analysis, PLS‐DA analysis, Permutation testing, OPLS‐DA analysis of the variability and reliability between samples from the ISO and KOA groups; (C) VIP analysis of differential metabolites between the KOA and SHAM groups; (D) VIP analysis of differential metabolites between the ISO and KOA groups; (E) KEGG topology analysis of differential metabolic pathways between KOA and SHAM groups; (F) KEGG topology analysis of differential metabolic pathways between ISO and KOA groups; (G) Differences in glycerophospholipid metabolism between KOA and SHAM groups; (H) Differences in glycerophospholipid metabolism between ISO and KOA groups.

## Data Availability

All results and data are maintained in the Department of Orthopaedics and Traumatology, Suzhou Hospital of Traditional Chinese Medicine, Suzhou, China. Data will be made available from the corresponding author on reasonable request.
