# Supplementary figures and images for: Isoimperatorin Reduces Synovial Inflammation and Fibrosis in Knee Osteoarthritis via the cAMP Signalling Pathway
Source: J Cell Mol Med. 2025 Oct 6;29(19):e70880. doi: 10.1111/jcmm.70880 (PMC12500418; doi:10.1111/jcmm.70880)

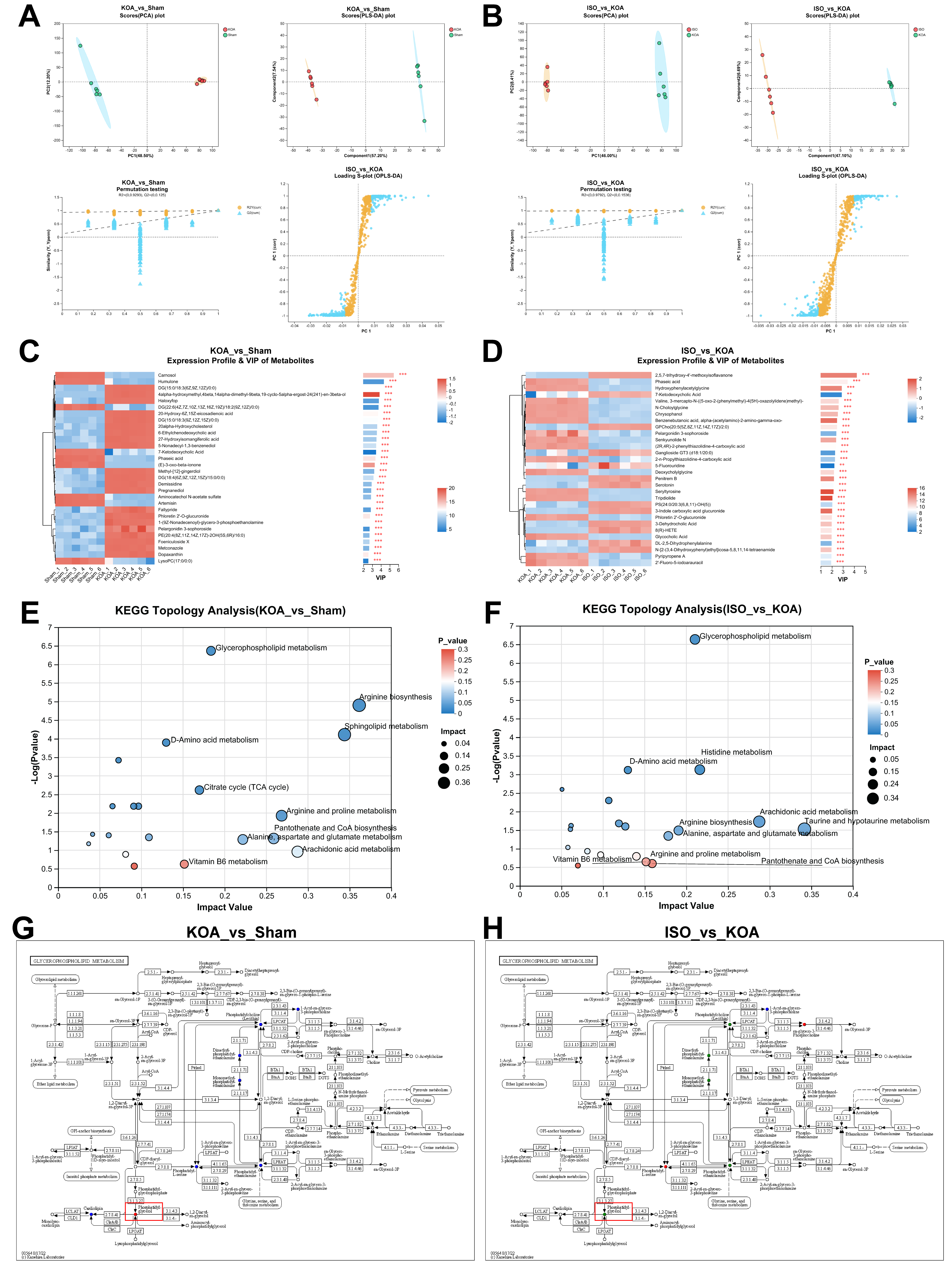

Supplement: Supplementary file 1 — FIGURE S1: (A) PCA analysis, PLS‐DA analysis, Permutation testing and OPLS‐DA to analyse the variability and reliability between samples from the KOA and SHAM groups; (B) PCA analysis, PLS‐DA analysis, Permutation testing, OPLS‐DA analysis of the variability and reliability between samples from the ISO and KOA groups; (C) VIP analysis of differential metabolites between the KOA and SHAM groups; (D) VIP analysis of differential metabolites between the ISO and KOA groups; (E) KEGG topology analysis of differential metabolic pathways between KOA and SHAM groups; (F) KEGG topology analysis of differential metabolic pathways between ISO and KOA groups; (G) Differences in glycerophospholipid metabolism between KOA and SHAM groups; (H) Differences in glycerophospholipid metabolism between ISO and KOA groups. [file JCMM-29-e70880-s001.tif]
